# Foreign Body Inside the Tunnel: A Rare Cause of Acute Cubital Tunnel Syndrome

**DOI:** 10.1055/s-0039-1696951

**Published:** 2019-10-28

**Authors:** Gokce Yildiran, Mustafa Sutcu, Osman Akdag, Zekeriya Tosun

**Affiliations:** 1 Department of Reconstructive and Aesthetic Surgery, Hand Surgery Division, Selcuk University Medical Faculty Plastic, Selcuklu, Konya, Turkey

**Keywords:** acute, cubital tunnel syndrome, foreign body

## Abstract

Foreign bodies are common entities found in hand surgery practice. However, they are a very rare cause of the acute cubital tunnel syndrome. A 48-year-old male patient was consulted for cubital tunnel symptoms after 2-day unconscious state in the intensive care unit. The ulnar nerve was explored, a piece of glass was removed inside the cubital tunnel, and the nerve was repaired. However, compression neuropathy symptoms due to the acute trauma are interesting. Nerve laceration with a foreign body should be considered in acute-onset cubital tunnel syndrome, in which the foreign body history of a trauma patient cannot be determined explicitly.

Peripheral nerve injuries of the upper extremity are leading causes of hand surgery emergencies. However, foreign bodies are one of the rare causes of these injuries. 1 Here, we present a case of a foreign body inside the cubital tunnel with acute cubital tunnel syndrome symptoms.

## Case

**Fig. 1:**
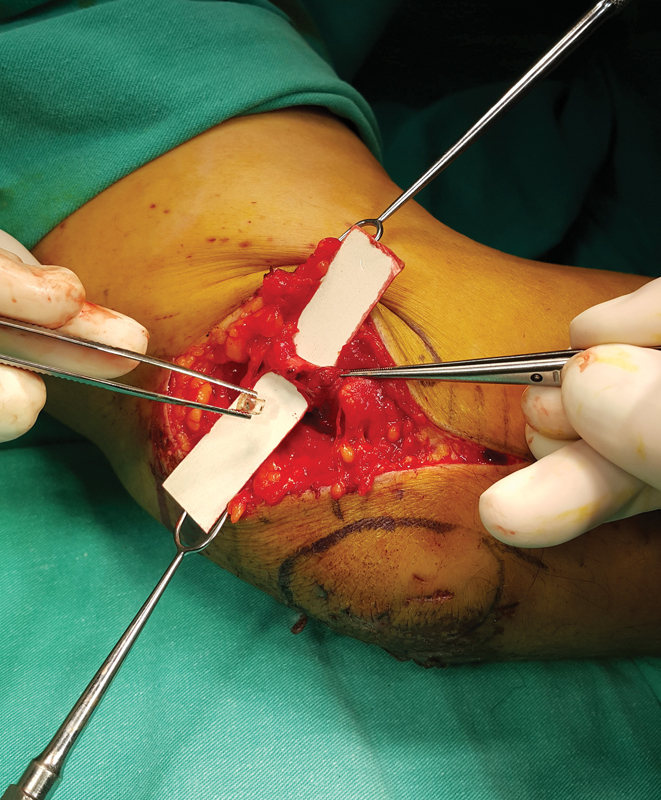
Intraoperative view shows the piece of glass inside the cubital tunnel and the laceration of the ulnar nerve.

A 48-year-old male patient presented with a traffic accident and was followed up in an unconscious state for 2 days in the intensive care unit of our hospital. After he became cooperative, we consulted him for paresthesia on the fourth and fifth fingers and the informed consent was taken. He had a small scar in the epicondylar region. Accordingly, conventional cubital tunnel incision was performed to explore the nerve, revealing a piece of glass inside the cubital tunnel and a partial laceration in the ulnar nerve ( Fig. 1 ). We removed the foreign body and repaired the nerve epineurally. Furthermore, a cast was applied, and physiotherapy was initiated for the patient. Patient was followed up with physical examination and the neurological function was fully recovered.

## Discussion

Although foreign bodies are common on the upper extremity, these rarely cause nerve damage. Choudhari et al reported a patient with progressive ulnar nerve dysfunction because of a foreign body migration. 1 Retained objects can cause nerve dysfunction even in the absence of a nerve laceration; however, owing to the granuloma, which they form around the nerve. In our case, the foreign body lacerated the nerve itself and, remarkably, it was inside the cubital tunnel. Pleser et al defined a foreign body located inside the ulnar nerve in the distal humerus. 2 Our case exhibited symptoms of acute cubital tunnel syndrome. In addition, some studies have reported venous thrombosis, hemangioma, and calcific neuritis resulting in acute cubital tunnel syndrome. 3 4 5 However, the compression neuropathy symptoms due to the acute trauma is interesting. It is known that the repetitive traumas and injuries are the main etiologies for the compression neuropathies. As a chronic disease, the compression neuropathies can be detected with nerve conduction studies; however, because being a trauma patient with the absolute indication for acute exploration and the degree of muscle denervation after nerve injury cannot be determined until Wallerian degeneration is completed (approximately after 4 weeks), we did not perform any preoperative electromyography study. 6 Notably, the determination of etiology that causes the syndrome for patients who are unconscious for a period, like our case, remains challenging. Hence, a detailed history should be obtained, and a comprehensive physical examination should be performed in cases such as ours.

Furthermore, nerve laceration with a foreign body should be considered in acute-onset cubital tunnel syndrome, in which the foreign body history of a trauma patient cannot be determined explicitly.
